# Making soft intelligence hard: a multi-site qualitative study of challenges relating to voice about safety concerns

**DOI:** 10.1136/bmjqs-2017-007579

**Published:** 2018-02-19

**Authors:** Graham P Martin, Emma-Louise Aveling, Anne Campbell, Carolyn Tarrant, Peter J Pronovost, Imogen Mitchell, Christian Dankers, David Bates, Mary Dixon-Woods

**Affiliations:** 1 Department of Health Sciences, University of Leicester, Leicester, UK; 2 TH Chan School of Public Health, Harvard University, Boston, Massachusetts, USA; 3 School of Pharmacy, Queen’s University Belfast, Belfast, UK; 4 UnitedHealthcare, Minnetonka, Minnesota, USA; 5 Australian National University Medical School, Canberra, Australia; 6 Department of Quality and Safety, Brigham and Women’s Hospital, Boston, Massachusetts, USA; 7 Division of General Internal Medicine, Brigham and Women’s Hospital, Boston, Massachusetts, USA; 8 Harvard Medical School, Boston, Massachusetts, USA; 9 THIS Institute, University of Cambridge, Cambridge, UK

**Keywords:** risk management, qualitative research, patient safety

## Abstract

**Background:**

Healthcare organisations often fail to harvest and make use of the ‘soft intelligence’ about safety and quality concerns held by their own personnel. We aimed to examine the role of formal channels in encouraging or inhibiting employee voice about concerns.

**Methods:**

Qualitative study involving personnel from three academic hospitals in two countries. Interviews were conducted with 165 participants from a wide range of occupational and professional backgrounds, including senior leaders and those from the sharp end of care. Data analysis was based on the constant comparative method.

**Results:**

Leaders reported that they valued employee voice; they identified formal organisational channels as a key route for the expression of concerns by employees. Formal channels and processes were designed to ensure fairness, account for all available evidence and achieve appropriate resolution. When processed through these formal systems, concerns were destined to become evidenced, formal and tractable to organisational intervention. But the way these systems operated meant that some concerns were never voiced. Participants were anxious about having to process their suspicions and concerns into hard evidentiary facts, and they feared being drawn into official procedures designed to allocate consequence. Anxiety about evidence and process was particularly relevant when the intelligence was especially ‘soft’—feelings or intuitions that were difficult to resolve into a coherent, compelling reconstruction of an incident or concern. Efforts to make soft intelligence hard thus risked creating ‘forbidden knowledge’: dangerous to know or share.

**Conclusions:**

The legal and bureaucratic considerations that govern formal channels for the voicing of concerns may, perversely, inhibit staff from speaking up. Leaders responsible for quality and safety should consider complementing formal mechanisms with alternative, informal opportunities for listening to concerns.

## Introduction

Healthcare systems have long grappled with the challenges of identifying, addressing and preventing problems of poor quality and safety.^1^ Information known to those working at the sharp end of care is increasingly recognised as an important resource in anticipating and preventing harm, but is often neglected.^2 3^ Sometimes this is because a tendency towards ‘comfort-seeking’ rather than ‘problem-sensing’ behaviours among leaders^4^ may result in personnel remaining silent or organisations failing to hear.^5^ Less well understood is why organisations fail to uncover concerns even when they are, in principle at least, eager to do so. Important challenges remain poorly understood in accessing ‘soft intelligence’: the kind of information known at the sharp end of care that characteristically escapes capture but may offer a valuable guide to potential problems.^2^

As in other industries, comprehensive insight into threats to safety will likely depend on employees at the sharp end giving voice to safety concerns.^6 7^ In the healthcare context, much research and policy attention has focused on the development of systems to enable access to safety relevant information.^8 9^ They include monitoring and surveillance of quality indicators,^10^ incident reporting systems and risk management techniques adopted from other industries.^11^ Policy interventions have sought to provide legal safeguards for whistle-blowing^12^ and encourage openness and transparency.^13 14^ These approaches have important strengths, but by themselves may not provide a complete picture; on occasion they may mislead.^8 15^

One reason why formal systems fail to surface the breadth of concerns is that speaking up is heavily influenced by cultural, psychological and social factors.^16^ For instance, incident reporting may be resisted by professional groups who resent managerial control and erosion of professional independence.^9^ Individuals in interdependent groups may be troubled about potential damage to relationships arising from challenging the status quo or calling into question others’ behaviour.^17^ Recent work has also identified the importance of implicit theories about the negative consequences of giving voice,^18^ and has deepened understanding of the influence of the type of concern (eg, safety vs professionalism) on speaking up.^19 20^

In this article, we describe a further possible influence on speaking up: how certain properties of formal channels for speaking up—including information technology-based mechanisms such as incident reporting systems, and also formal policies and protocols for raising concerns through the managerial hierarchy—may, perversely, act as deterrents to voice.

## Methods

We conducted a qualitative study involving semistructured interviews in three healthcare organisations. The selection of sites was initially pragmatic: one organisation, having experienced a serious problem involving patient harm, commissioned a study to understand how to improve voice by examining practices of speaking and listening within its hospitals. Following initial data collection and analysis, two other sites were chosen purposefully to extend the analysis and to test the transferability of constructs to other contexts: one organisation, in the same country as the first and with some similar characteristics (a prestigious teaching hospital), had undertaken a programme of cultural enhancement that included a focus on practices of voice; the other was in a different high-income country. All three were relatively large organisations based across several sites, and all three were academic medical centres with affiliations with nearby university medical schools.

In each site, heads of purposively selected departments were asked to distribute an email asking colleagues to participate in a confidential interview. With a view to ensuring that the views of a range of occupational groups were included in the study, we sought to include leaders and managers at the blunt end of care, and individuals at the sharp end (eg, physicians, nurses, technical/administrative staff, buildings and housekeeping staff). The email included an information brochure explaining the study, stressing its confidential nature and guaranteeing that no one at the hospital would be told who had participated. The email included a link to a confidential response website. Interested individuals provided contact details on this website, were contacted by an interviewer and given further information. Arrangements were made to conduct a telephone interview with those still interested in participating.

Semistructured interviews were conducted using a topic guide that included questions about how personnel raised concerns about situations or practices that they felt might not support patient safety. All interviews were digitally audio-recorded. At site 1, interviews were conducted by GM and a freelance interviewer; interviews at other sites were conducted by ELA. Care was taken to ensure independence of the data collection and analysis process. Data collection and transcript coding were undertaken by researchers with no connection to the case study organisations; interview transcripts were not shared with any staff in the organisations. Although the initial study was commissioned by the first hospital, freedom to publish findings was agreed from the start.

This study was submitted for ethical review at each participating organisation. At two sites it received approval. At one site it was deemed quality improvement and exempted from approval; the study team nonetheless used a consent procedure at all sites. Interview recordings were transcribed verbatim, and transcripts were anonymised. Following transcription, recordings were deleted. No link was retained between transcripts and participants. Transcripts were not shared with any personnel at participating hospitals.

Data analysis was based on the constant comparative method.^21^ A selection of interviews was open coded to develop an initial coding frame which was applied to subsequent transcripts, and iteratively refined as new codes were defined. NVivo software was used to manage the process.

In presenting our findings, we occasionally alter minor details of quotations to preserve anonymity.

## Results

We received 329 initial responses to the invitation for interview and conducted 165 interviews (table 1). We did not sample among those who responded, instead interviewing everyone who both responded and was able to make arrangements for an interview. Across participants, there was acknowledgement of the importance of concern raising as a means of ensuring vigilance about quality and safety, but while more senior participants often drew attention to the role of formal channels, it was clear that those at the sharp end had many anxieties about these mechanisms. We explore these views, and their consequences for the effectiveness of the organisations’ efforts to promote voice, in the five sections that follow.

**Table 1:** Responses to invitation and interviews conducted across the three sites

	Responses to invitation	Interviews conducted	Interviews with leaders and senior managers: the ‘blunt end’	Interviews with frontline personnel: the ‘sharp end’
Site 1	118	67	20	47
Site 2	78	47	16	31
Site 3	133	51	21	30
Total	329	165	57	108

### Valuing voice

In interviews, leaders and senior managers across the sites emphasised the need for concerns about safety and quality to be raised by personnel throughout the organisation, at every level. Many stressed that they welcomed information about anything with potential consequence for safety, whether technical, systemic or behavioural, and regardless of apparent gravity. In no site was there a shortage of mechanisms for people to report incidents or raise concerns, from online incident reporting systems to staff surveys and morbidity and mortality conferences, as well as encouragement to confide in senior personnel.

I just tell every employee during orientation that they can always feel comfortable coming to me if they have concerns that they want to share. (Executive, Site 1)

I’m pretty liberal about safety reports. I encourage them to do them no matter what, even if it seems minor. (Director, Site 3)

Across the sites, however, participants reported a pervasive sense that much potentially relevant intelligence did not reach managerial level and was not detectable through these formally instituted systems.

I do believe that we have a safe environment where people feel safe to express their concerns. But again we are human and there are times where, depending on the stakes involved and the conversation, sometimes there is some hesitancy. (Manager, Site 1)

Everybody pretty much keeps to themselves, or if they feel a certain way about something they will share it with another co-worker and they’ll bicker, but the problem is not going to be addressed because it’s just gossip. Not getting to the core that can actually do something about it. (Clerk, Site 1)

Some reasons given for the obscuring of such concerns echoed the existing literature on speaking up: the personal effort and risk associated with raising issues, lack of feedback and a sense of futility. As they are widely reported in the literature,^22–26^ we do not repeat them here. Instead, we focus on the paradoxically silencing effect of formal systems.

### The logic of formal systems

As well as reporting their enthusiasm for voice, many senior leaders described an instinct towards formalisation. To make a concern knowable at the blunt end^27^ (senior/executive level) meant converting it into a form recognisable as legitimate evidence. Soft intelligence needed to be made ‘hard’: properly documented, formalised and amenable to verification. For leaders, the simplest way of achieving this was to encourage people to use one of the existing systems, rendering the concern as something that could reasonably be known and in a recognisable form.

[If] one of the receptionists comes up and says, ‘This thing happened with this person and that wasn’t right and I’m afraid it’s going to happen again’—the pretty much universal response for that now is, ‘Yes, that is a concern; fill out [an incident report].’ (Medical director, Site 1)

Regardless of the form or shape in which a concern surfaced, formal channels were geared towards a goal of establishing facts through defined procedures. In their accounts of establishing ‘the facts’ of a given situation, leaders emphasised respect for due process and quality of evidence. They explained how investigating concerns could involve a painstaking process of disambiguation, involving much effort and regard for fairness. For example, the need to understand the different ‘sides’ of a story was frequently invoked.

I usually interview all the staff, I collect all the information and I tell the manager I’m going to do this to understand what the issues are. Because I know it’s always two-sided: I want to hear what the manager’s perspective is about the issue but I want to hear each individual staff member. (Senior leader, Site 1)

I would ask her to document her recollection of the events. If she doesn’t feel comfortable I would tell her that I was going to take some notes. I would take some notes, get her feedback, say to her, ‘Is this what you’re telling me?’ Paraphrasing what she’d said, and make sure she agrees with what I’ve documented. Then I’d get the staff member in and say, ‘Can you tell me about this incident that happened with this patient on this day?’ Get his take on it. (Director of nursing, Site 2)

Also important was an interest in defensibility. Leaders sought to minimise exposure to risks of litigation, complaints of bullying or discrimination, or union action.

You have to meet with the staff, and they have a representative, and you do, and then it’s ‘He said’, ‘She said’, right? Because unless I’m there standing watching it, unless someone’s sent me an e-mail with all of the information, or documented it, I’m stuck because at the end of the day they’re going to deny it. (Senior nurse, Site 3)

Often there is a counterclaim of bullying/harassment made, that is a vexatious claim made by someone because they don’t like how the person operates. So there is a lot of this nonsense that goes on. (Manager, Site 2)

Attention to due process satisfied administrative and legal requirements for fairness, meticulousness of procedure and the ability to justify decisions and actions. But it also had perverse effects. These included, most notably, the potential to stifle sharing of information and suppress soft intelligence, turning it into a form of ‘forbidden knowledge’^28^ that was dangerous to know or reveal.

### The keeping of secrets

In interviews, it became clear that some concerns were never voiced, notwithstanding the plethora of available mechanisms.

I think [staff are] fairly comfortable on [raising] factual things, like the medication came up with the wrong dose. Completely uncomfortable on personal-behaviour stuff. That would never, never, [arise] on a safety round, I can tell you that. (Professor, Site 1)

In part, this reluctance was due to generalised concern and uncertainty about the potential consequences of such an act, especially when it meant indicting a colleague. Participants were much happier to use formal channels to report concerns about systems or equipment failures, where individual competence or behaviour was not implicated.^20^ Central to reluctance to speak up about concerns about individuals was the widely shared understanding that to raise a concern formally was to allow it to pass from one world (that of the sharp end) to another (the blunt end, with its processes and procedures). In that other, blunt-end world, incipient concerns would be processed through a set of bureaucratic controls aimed at making them hard: evidenced, formal, tractable to organisational intervention. Inchoate intuitions or suspicions would be expected to demonstrate some degree of orderliness; conflicts over the proper definition of particular situations would be adjudicated.

There’s a lot of weight that goes into that, and what if my assumption was wrong? […] I would go to someone first, would talk it through first. (Nurse, Site 3)

And you just feel like you’re taking this piece that feels like a more visceral experience, and trying to parse it into these artificial structures that have no emotional weight for you. (Attending physician, Site 3)

Unsurprisingly, participants’ doubts about giving voice often related to their confidence that they could demonstrate the validity of their concerns. When they were unsure that the concerns could survive harsh scrutiny, they were likely to maintain silence. For one thing, they did not want to become drawn into a wearying cataloguing of evidentiary artefacts that would legitimise their claims.

The next step for me was to start documenting specific behaviours, and have like ‘On 8 December this person did this at 10 o’clock,’ so I would have evidence of what was happening. (Allied health professional, Site 2)

Further, to voice a concern that passed into the world of the blunt end was not only to allow it to be formalised, but also to have a share in the consequences. Participants sometimes saw the formal procedures followed to establish the ‘facts of the matter’ as risking an escalation of hostilities that would ultimately undermine other, more important goals—for example, preserving good working relationships—or that would erupt into an outcome disproportionate to the circumstances.

Depending on what the circumstance is—I don’t want to be responsible, potentially, for someone getting fired or something catastrophic to happen. (Registered nurse, Site 1)

Anxiety about evidence and process was particularly relevant when the intelligence was especially soft—feelings or intuitions that were difficult to resolve into a coherent, compelling reconstruction of an incident.

If I’m not perfectly sure of my standing on a certain issue, I have to do some real homework to really feel like I’m in charge and then I can feel more comfortable. But I might feel uncomfortable if I don’t know all the facts or if I don’t know the heart of the story. (Administrator, Site 1)

Formal systems in the sites were geared towards resolution—by rectifying a system problem, improving a clinical process or addressing a behavioural issue—or, conversely, exonerating an individual or team of culpability. Senior leaders’ descriptions of how they would respond to a formally raised concern typically included a plan of action designed to tackle the problem as an endpoint. Sometimes this was appropriate. But for these more ambiguous problems, this orientation towards resolution could deter those with concerns from broaching them formally, because—in their view—the issue was simply too ambiguous, too complex, too unformed to be amenable to resolution. Participants recognised the limitations of even the most thorough of investigations, and therefore worried that any intervention might be premature, disproportionate or misdirected.

Have I ever [raised concerns formally]? No, I guess not really. […] I mean for all I know maybe they were so short-staffed that person could, maybe the order never got to this person to draw [a blood]. (Physician, Site 3)

I think that it judges the person who we are putting in, or who we are mentioning, unnecessarily […] rather than we just want to raise and it needs to be looked into. (Physician, Site 2)

### Informal validation of concerns

Participants described informal sense-checking, fact-finding and behaviour-monitoring as a routine part of work at the sharp end. It formed a functional part of the day-to-day regulation of behaviour, particularly among clinical peers, allowing concerns to be addressed in situ without engaging formal systems. But participants also noted its downsides. In particular, once a norm of dealing with problems locally became dominant, it could lead to implicit tolerance for behaviours that were unacceptable.

I will voice my issues with friends, like if I go to lunch or something, as long as it is not something that is confidential. I have a confidante here that I will throw things back and forth at. […] I will get other people’s opinions on how to handle things. I never have an issue with that. I think the more the merrier when you are trying to fix something that really needs to be fixed. (Supervisor, Site 1)

There was a lot of talk about [competence issue] before anything was ever done to address it with her, in a formal way at least. People pointed out her errors along way, in terms of what she should have done to be more prepared, how she needed to be more thorough. But I do think there was a certain amount of hesitancy to do anything formal about it, because of knowing that’s someone’s career. (Attending physician, Site 3)

Where individuals felt that intervention was needed, one strategy was to engage informal validation processes that had some symmetries with formal approaches to establishing facts. Participants described corroborating their story with others and gaining allies. In these situations, they built up portfolios of evidence and sought safety in numbers through collective voice.

A co-resident and I were sent off to the gastroenterology clinic for an afternoon, and we were both pretty sure that the attending was drunk. We noticed it independently, that he smelt like alcohol. And we spent like three days talking to each other about what should we do. We really didn’t know what we should do. And ultimately we went to talk to our programme director. (Primary care physician, Site 3)

### Accessing soft intelligence

Some managerial participants described specific proactive strategies for supplementing formal systems with other sources of knowledge. These strategies had two defining characteristics. First, they sought to separate *gathering* intelligence from *acting on* intelligence. What seemed important was creating opportunities for voice without simultaneously imposing the expectation that a formal process would necessarily follow.

It might be one colleague coming to me and saying, ‘Did you know so-and-so, this is happening with this individual.’ So there are lot of ways, I don’t want to call them informants, but there are a lot of ways that the staff feel comfortable bringing things to us. (Senior leader, Site 1)

Second, these strategies were explicitly *relational*: they involved leaders being visible and available, and seeking to create trusting relationships where colleagues could feel confident about raising sensitive or embryonic concerns. When leaders—at all levels, but particularly in senior clinical roles—took steps to meet sharp-end colleagues and make personal connections, they were more likely to hear concerns.

We have this thing called coffee with nurses. […] So when it is a sit-down talk about patient safety, it seems like those things kind of get stilted. Whereas what in my opinion has made conversations about difficult issues easier has been now that I have been here long enough. And having those interactions, […] when the time comes to have a difficult conversation, then they can take place without being under the spotlight. (Physician, Site 1)

Skilful, supportive middle managers were identified as having an important role in encouraging staff to voice concerns: opportunities for discreet conversations about sensitive issues were especially valued. Central to perceived success was shedding the trappings of formal processes to create environments where people felt comfortable in raising concerns, and confident that they would be dealt with proportionately and appropriately.

She is not saying, ‘Well that is completely stupid,’ or ‘Have you lost your mind?’ or anything like that. She says, ‘Let’s look at this objectively,’ and then if it turns into something that needs to go to a different department or further up the command structure, she is the one that takes it up there and she says, ‘My people have identified this as an issue.’ (Administrator, Site 1)

## Discussion

This study, involving a large number of interviews with organisational participants at multiple levels across three sites, suggests that some potentially relevant intelligence may never reach managerial level, and is not detectable through formally constituted systems for raising concerns. Instead, it remains fugitive, part of a hidden world of confidences and half-secrets. Formal systems may, ironically, sometimes contribute to the subduing of voice, perhaps especially when it relates to concerns about colleagues’ competence, attitudes or behaviour. Participants at all levels were sensitive to the logic of action that follows from formalisation of concerns: once concerns pass from sharp to blunt end, they are in the domain of the system, where certain bureaucratically ordained processes must follow, and where downstream consequences are unpredictable. This results in material and emotional burden for those who voice concerns: they must evidence their concerns according to the formal criteria required by the blunt end, and they also become implicated in the consequences of voice for all parties, which are not predictable and may be profound.

The bureaucratic form taken by formal channels exists not because those at the blunt end are insensitive to the complexities of clinical and emotional realities. Rather, it is because of the demands of legal and regulatory frameworks, institutional norms and organisational policy. But the urge for certainty and resolution does not sit comfortably with many of the concerns that reside at the sharp end. Here, people worry that efforts to clarify might be premature, and result in inappropriate or even counterproductive outcomes. The risk, then, is that attempting to find the ‘facts of the matter’ through proceduralised processes—turning the soft into hard—might actually result in information losses rather than gains. The result is that potentially valuable information about risks to patient safety becomes a form of ‘forbidden knowledge’,^28^ suppressed through social and cultural pressures. For those who see in employee voice a route to organisational vigilance and early intervention to prevent harm,^2 5 29^ this insight poses important challenges.

The emphasis in managerial accounts on due process, procedure and establishing facts that we have found is not, of course, unique to healthcare. It is consistent with a long-term, widespread trend towards *legalism*—where legal considerations and a concern for defensibility play an increasingly central role in organisational life.^30^ A key consequence of legalism is that, as Michael Power notes, ‘organizations must make themselves auditable and present their operations in specific ways which are aligned with legalized culture.’^31^ The move towards legalism has many positive effects, including respect for due process, considerations of equity and fairness and discouragement of vexatious complaints. But it is not wholly benign. Building on previous work,^5^ we suggest that a preoccupation with formal systems may lead to neglect of other expressions of concern, including the wealth of informal, interpersonal ways individuals identify and manage problems every day.^20 32^

This leads us to the question of how to harvest soft intelligence of the kind that might be valuable to leaders. The solutions are unlikely to lie in designing ever more elaborate systems for reporting concerns: as Sitkin and Roth argue,^33^ additional formal mechanisms may simply lead to an ‘arms race’ of systems that do little to address the underlying problem and that further erode trust:

The adoption of legalistic ‘remedies’ (ie, institutionalized mechanisms that mimic legal forms and exceed legal/regulatory requirements) imposes a psychological and/or an interactional barrier between the two parties that stimulates an escalating spiral of formality and distance and leads to a need for more rules.

Our findings similarly suggest that the nature of formal processes and the bureaucratic systems in which they are ensnared means that no amount of effort to improve such processes, for example, by making them more accessible, exhorting individuals to report concerns or emphasising that they exist for learning rather than blame, will fully overcome this challenge. This is not, of course, to suggest that there is no place for such systems: they are vital. Nor is it to suggest that these systems cannot be improved. But for certain kinds of problems, some of which may be critical to foresight and prevention of harm, they may not provide a full solution. With this in mind, we offer two possible routes to restoring the connection between blunt end and sharp end and permitting the sharing of inchoate yet potentially critical insights.

One is that informal, peer-oriented use of voice in response to concerning practice and behaviour may be an effective, low-cost way of handling possible breaches of standards of good practice or conduct.^34^ Leaders might usefully recognise that not all voice behaviours need result in formalisation or action; it may be more helpful to support local problem-solving, while ensuring that an escalation plan is available should this fail, and that problems are not tolerated for too long. A particular risk here is that leaders immersed local cultures might fail to recognise the problems associated with entrenched behaviours; any such approach will need to be accompanied by clear statements of appropriate standards of conduct. Such an approach is also likely to require new skills (including those relating to listening and counselling) and new norms (including curbing the urge to intervene formally).

A second solution is relational in character. There were hints in our data that leaders who were visible, trustworthy and provided informal opportunities to listen may have some success in improving voice behaviour. Crucially, this meant abandoning the quest for clarity and certainty, and accepting the ambiguity inherent in such signals, avoiding a rush to action that may be inappropriate, premature or have unintended consequences. The extent to which senior leaders are able to devote time to such activities in resource-constrained healthcare organisations will vary; it is also important that opportunities for informal discussions are governed by trust and confidence, rather than being seen as an exercise in accountability.^35^

Our study has limitations. It is based on interviews only, and we had no means of verifying behaviours, practices or impacts. While we did all we could to emphasise confidentiality and make participation as safe as possible, we cannot say whether this was successful in securing participation of a breadth of informants, or in obtaining complete accounts from interviewees. Finally, while the number of participants was large for a qualitative study, data collection was limited to three institutions (each with a strong focus on improving its safety culture) in two high-income countries; this may limit its transferability beyond such settings, though it is plausible that the challenges we found would be at least as acute in institutions less intent on improving voice. All three organisations were also large, academic medical centres; in other organisations (including smaller acute hospitals, and also other healthcare settings where the gap between blunt end and sharp end is less pronounced, where staff are fewer in number, and where interpersonal and interprofessional relationships may be rather different) our findings may have been quite different.

## Conclusion

Our study suggests that on occasion, efforts to glean insights from the sharp end about risks to quality and safety may be thwarted by the very mechanisms intended to facilitate communication. The nature of concerns about quality and safety means that they are often partial, incomplete and ill formed. Systems that demand clarity and certainty, whether because of well-meaning regard for due process and evidential foundation or the dominance of legalistic processes in organisations, may deter those at the sharp end from voicing such concerns. If organisations value these insights as a means of sensing problems proactively, they may require other approaches to accessing them that are less pervaded by formality and the search for certainty.
